# Above-Knee Amputation After Total Knee Replacement Infection: The Unfortunate End of an Odyssey

**DOI:** 10.7759/cureus.35052

**Published:** 2023-02-16

**Authors:** Ioannis Savvidis, Dimitrios Rigkos, Evaggelos Solovos, Dimitrios Georgiannos, Ilias Bisbinas

**Affiliations:** 1 1st Trauma and Orthopaedics Department, 424 General Military Training Hospital, Thessaloniki, GRC

**Keywords:** reemergence of infection, knee arthrodesis, above knee amputation, total knee replacement, periprosthetic joint infection

## Abstract

Total knee replacement is the gold standard for the surgical treatment of late-stage symptomatic knee osteoarthritis when conservative measures have not alleviated the problem. However, as with any surgery, there are potential dangers and complications. Of these, infection is one of the most severe and may lead to life-changing outcomes for the patient. In this case report, a patient with a history of infected primary total knee arthroplasty and numerous attempts to eradicate the patient’s infection is presented. After two unsuccessful two-stage revisions, and although arthrodesis was discussed and suggested, an above-knee amputation was finally applied to our patient.

## Introduction

Total knee replacement (TKR) is an increasingly attractive treatment for degenerative knee disease, with approximately 90% improvement in the patient’s condition [1]. Nevertheless, as with any operation, TKR might be associated with complications [2]. Relapse or persistence of the periprosthetic joint infection (PJI) is one of the most challenging possible complications that can occur up to 10% after a primary TKR [1,3]. Treatment strategies for PJI may include long-lasting antibiotic therapy, knee joint arthrodesis (KA), or above-the-knee amputation (AKA) [3]. There is no consensus about how many revisions can be performed. Is an arthrodesis or an amputation preferable after unsuccessful treatment of PJI [4]? In this case report we present a patient's odyssey, which culminated in an above-the-knee amputation after multiple attempts to treat the infection of his primary TKR.

## Case presentation

**Figure 1:**
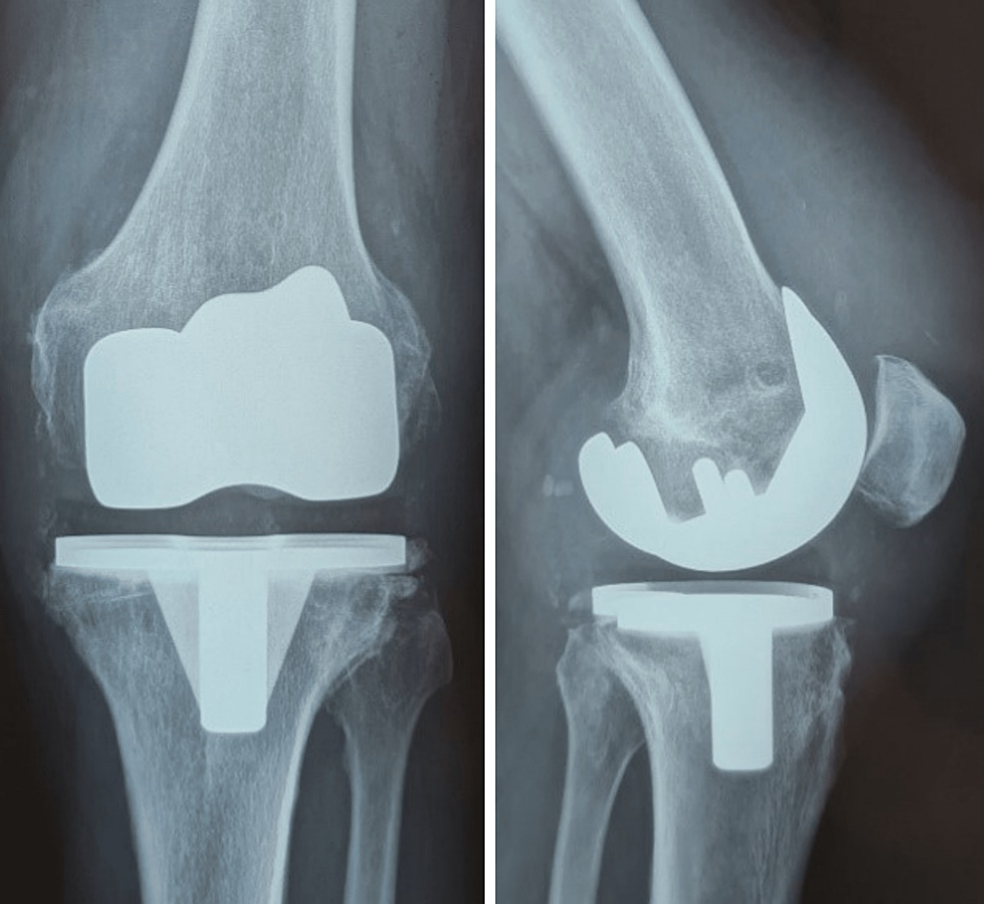
Antero-posterior (AP) and lateral (L) knee radiograph of the primary TKR

A 48-year-old male patient, a heavy smoker, without any known comorbidities, underwent a primary TKR due to posttraumatic knee osteoarthritis. (Figure 1). In his past surgical history, 15 years prior to his primary TKR he had a comminuted tibial plateau fracture in the same knee, which was treated with open reduction and internal fixation (plate and screws), and the metalwork was removed in a second operation two years later, thirteen years prior to his TKR.

**Figure 2:**
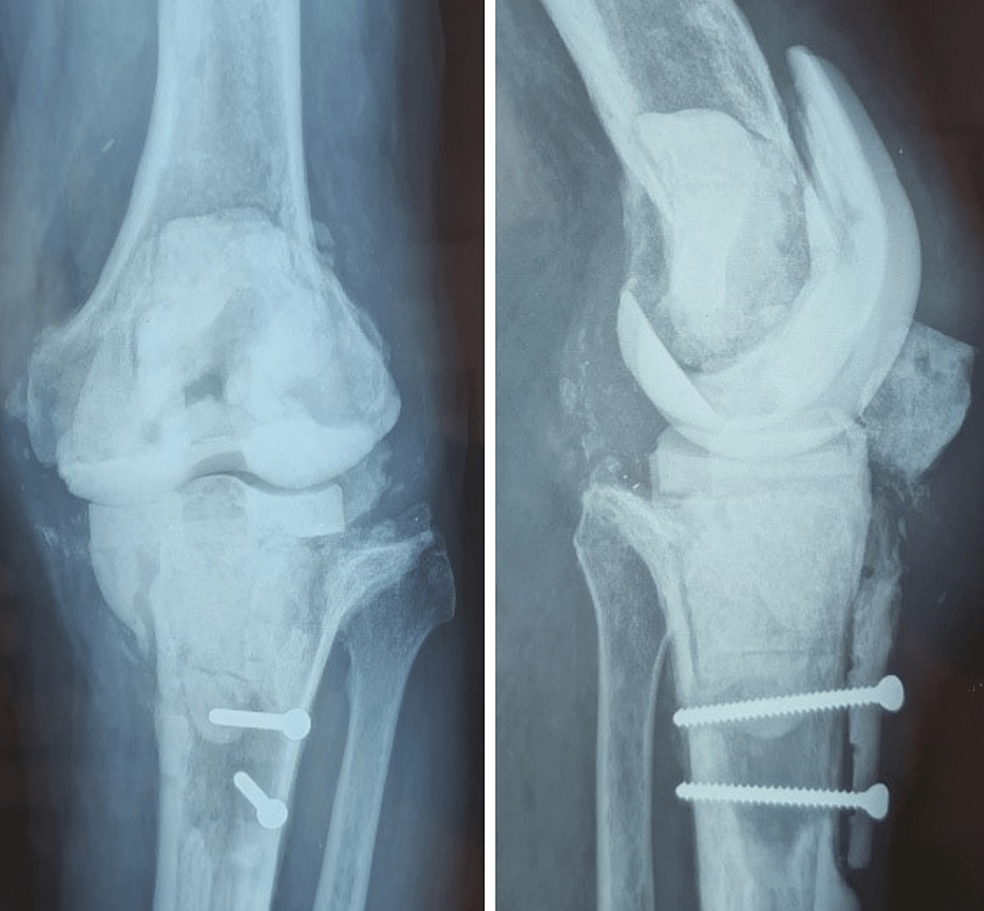
Antero-posterior (AP) and lateral (L) knee radiographs of the first stage revision of TKR

**Figure 3:**
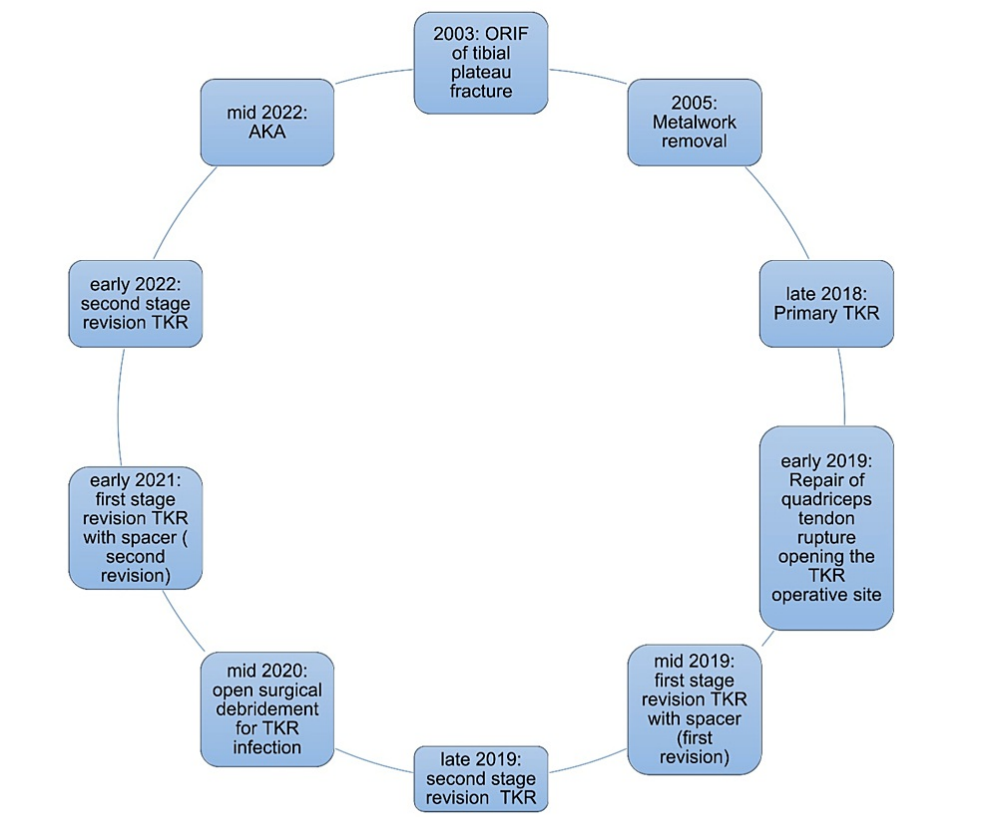
Patient's surgical history

Two months after his primary TKR, while in the recovery stage, he slipped, fell, and suffered a traumatic rupture of the quadriceps tendon of his freshly operated knee, which was treated with a surgical repair of the tendon opening at the previous operative site. Six weeks after his tendon repair, a PJI developed redness, pain, stiffness, and swelling around the knee joint. Synovial fluid cultures revealed a methicillin-resistant Staphylococcus epidermidis (MRSE) infection of the TKR. The same *MRSE* microorganism followed our patient on his journey until his AKA. He had two operations because of comminuted tibial plateau fracture, primary TKR because of secondary OA, quadriceps tendon rupture-repair, first two-stage revision because of PJI, surgical debridement, second two-stage revision because of PJI (Figure 2) relapse, and finally AKA (Figure 3). Although the option of KA was discussed with him, in between and after the two revision procedures, it was concluded that AKA was the best option for the patient. The patient underwent an AKA four years after his first TKR. In total, the patient had eight substantial surgical procedures over the past four years, had over five months of intermittent hospitalizations, and completed almost 13 months of antibiotic treatment (intravenous and orally) (Table 1). In addition, he had almost four years of rehabilitation and over 180 sessions of physiotherapy. It should be noted that during the last six weeks prior to his AKA, he developed signs of depression and needed psychiatric support.

**Table 1:** Antibiotic treatment after each surgery

Operation	Antibiotics Used	Duration
Quadriceps Tendon Repair	Ciprofloxacin – Rifampicin	1 month
First Stage Revision	Spacer ( Vancomycin – Gentamicin), Daptomycin – Dalbavancin (a month) Ciprofloxacin – Rifampicin (three months)	4 months
Second Stage Revision	Ciprofloxacin – Rifampicin	1 month
Open Debridement	Ciprofloxacin – Rifampicin	2 months
First Stage Revision	Spacer ( Vancomycin – Gentamicin), Daptomycin – Dalbavancin (a month) Ciprofloxacin – Rifampicin (three months)	4 months
Second Stage Revision	Ciprofloxacin – Rifampicin	1 month

**Figure 4:**
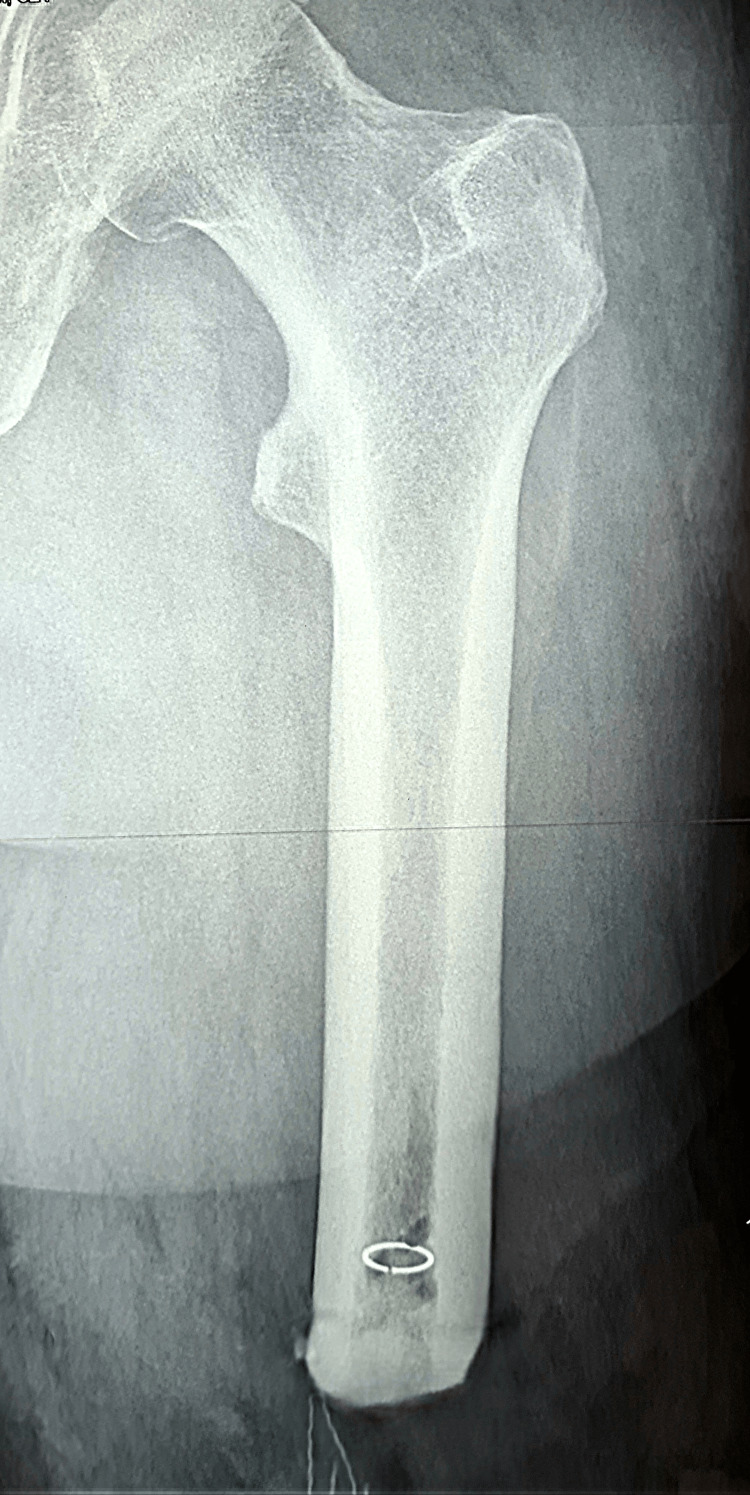
Antero-posterior (AP) radiograph of the AKA

A KA was discussed thoroughly with the patient as a viable option after his PJI relapse. However, he was reluctant to have an arthrodesis and therefore he opted for the two-stage revision TKR twice. After the second PJI relapse and because of physical and mental exhaustion, he developed signs of secondary depression, and he started receiving psychological support. He already had a long journey and the patient felt that it was time for a more permanent solution and therefore he opted for an AKA. Many factors had to be taken into consideration for the decision-making. Those included persistent chronic PJI, patient's age, his physical and mental exhaustion, multiple operations - soft tissue and bone loss, lower limb performance after KA or AKA and last but not least the fact that he had substantial difficulties to quit smoking. We explained to our patient that knee fusion would take up to two years to succeed, it might fail and need further operations as well as the fact that the infection after all might still not be eradicated. On the other hand, with AKA, although it is “one-way ticket” and psychologically the burden is heavier, quite likely he would not have any further hospital treatment and he would be back to his everyday activities possibly with a better functional outcome. Considering his long journey as well as his mental and physical exhaustion, he opted for an AKA (Figure 4).

**Figure 5:**
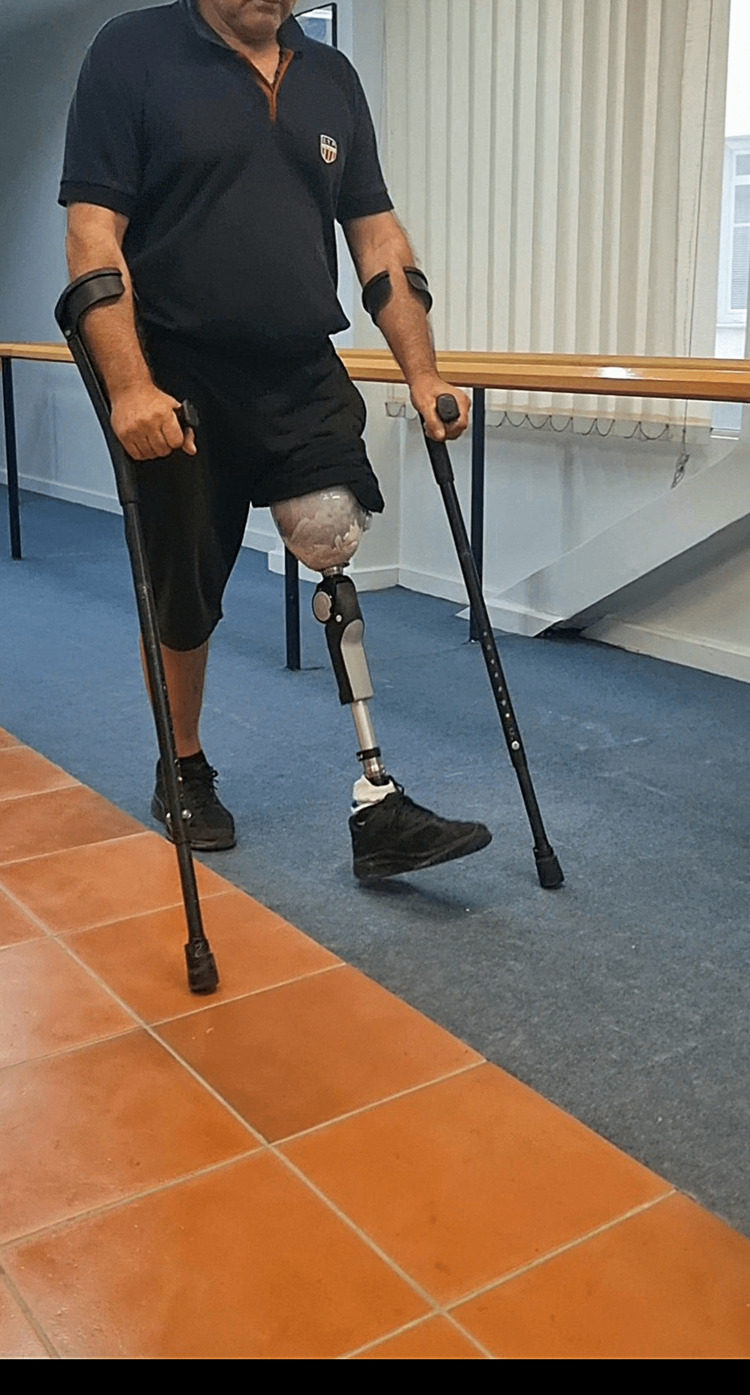
Photograph of the patient ambulating with a prosthesis and crutches

Four months after his AKA, he is clinically well, ambulating with a prosthesis and crutches (Figure 5). His inflammatory markers are within normal limits. His mental and physical condition has improved dramatically.

## Discussion

TKR is considered a highly successful surgery for the treatment of severe knee osteoarthritis with a success rate of around 90% [1]. TKR is one of the most effective operations of the last 50 years [2]. This procedure alleviates knee pain from OA and improves limb function and quality of life [1]. Although routinely, patient selection, appropriate preoperative preparation, strict anti-infection theatre rules, as well as prophylactic antibiotics preoperatively and postoperatively are being used, PJI is encountered in almost 2% of all knee arthroplasties [5]. PJI results in a considerable loss of life quality and physical and psychological stress for the patient as well as a significant financial burden on the healthcare system. It is estimated that by the year 2030, more than 3 million TKRs will be performed annually. Consequently, the number of revisions TKR procedures will increase as well, including those for TKR PJI [6]. The infection rate of either 1-stage or 2-stage revision for an infected TKR is calculated to be around 18% and 10%, respectively [7]. There are two available salvage procedures after primary TKR infection and PJI relapse after a two-stage revision: KA and AKA. After an infected primary TKR, there is a 0.025% amputation rate; however, it can increase to 5.1% after persistent infection despite revision TKR surgery. *Staphylococcus aureus* and *Staphylococcus epidermidis* are the most commonly isolated microorganisms for PJI and they are often quite resistant to multiple antibiotics [1,8]. Our patient had multiple procedures, some prior and quite a few after his primary TKR, and a long journey that took its toll both physically and mentally. Both Wu et al. and Robinson et al. concluded that KA is a more effective procedure after an infected two-stage revision TKR than another two-stage revision [4,5]. According to our experience, our patient’s odyssey would be quite likely shorter with a comparable final outcome if the patient had a knee arthrodesis instead of the second two-stage revision surgery and that agrees with the previous authors’ statement. Having multiple predisposing factors, KA would be better suggested early after the first revision and relapse of PJI, rather than after two more substantial and heavy operations (a second two-stage revision surgery) [9].

## Conclusions

Periprosthetic joint infection after a TKR is a challenge for both the patient and the surgeon. We present a patient with a nineteen-year journey, with two operations relative to a knee fracture, a TKR 15 years later, a tendon repair in the knee joint (early postoperatively after TKR), twice two-stage revisions because of PJI development and relapse, and one surgical debridement between them. All this long journey ended with an AKA four years after his primary TKR. Given the circumstances and experiencing all our patient’s journeys up to the present time, it is not clear whether we should proceed either to a KA or an AKA after his PJI relapse. Which option could be better, remains to be proved after more extensive research and randomized control studies. Subjectively, we believe that after the first two-stage revision for a PJI relapse, knee arthrodesis is a safer option, and it could always leave the option for an AKA if it is needed in a later stage.
